# Hypoxic Preconditioning Enhances Survival and Proangiogenic Capacity of Human First Trimester Chorionic Villus-Derived Mesenchymal Stem Cells for Fetal Tissue Engineering

**DOI:** 10.1155/2019/9695239

**Published:** 2019-11-12

**Authors:** Dake Hao, Chuanchao He, Bowen Ma, Lee Lankford, Lizette Reynaga, Diana L. Farmer, Fuzheng Guo, Aijun Wang

**Affiliations:** ^1^Surgical Bioengineering Laboratory, Department of Surgery, School of Medicine, University of California Davis, Sacramento, CA 95817, USA; ^2^Institute for Pediatric Regenerative Medicine, Shriners Hospitals for Children, Sacramento, CA 95817, USA; ^3^Department of Biomedical Engineering, University of California Davis, Davis, CA 95616, USA

## Abstract

Prenatal stem cell-based regenerative therapies have progressed substantially and have been demonstrated as effective treatment options for fetal diseases that were previously deemed untreatable. Due to immunoregulatory properties, self-renewal capacity, and multilineage potential, autologous human placental chorionic villus-derived mesenchymal stromal cells (CV-MSCs) are an attractive cell source for fetal regenerative therapies. However, as a general issue for MSC transplantation, the poor survival and engraftment is a major challenge of the application of MSCs. Particularly for the fetal transplantation of CV-MSCs in the naturally hypoxic fetal environment, improving the survival and engraftment of CV-MSCs is critically important. Hypoxic preconditioning (HP) is an effective priming approach to protect stem cells from ischemic damage. In this study, we developed an optimal HP protocol to enhance the survival and proangiogenic capacity of CV-MSCs for improving clinical outcomes in fetal applications. Total cell number, DNA quantification, nuclear area test, and cell viability test showed HP significantly protected CV-MSCs from ischemic damage. Flow cytometry analysis confirmed HP did not alter the immunophenotype of CV-MSCs. Caspase-3, MTS, and Western blot analysis showed HP significantly reduced the apoptosis of CV-MSCs under ischemic stimulus via the activation of the AKT signaling pathway that was related to cell survival. ELISA results showed HP significantly enhanced the secretion of vascular endothelial growth factor (VEGF) and hepatocyte growth factor (HGF) by CV-MSCs under an ischemic stimulus. We also found that the environmental nutrition level was critical for the release of brain-derived neurotrophic factor (BDNF). The angiogenesis assay results showed HP-primed CV-MSCs could significantly enhance endothelial cell (EC) proliferation, migration, and tube formation. Consequently, HP is a promising strategy to increase the tolerance of CV-MSCs to ischemia and improve their therapeutic efficacy in fetal clinical applications.

## 1. Introduction

Over the past three decades, with the development and exciting advances of fetal surgery for treatment of congenital diseases, fetal tissue engineering has been established as an emerging field of fetal medicine to augment *in utero* surgical approaches [1–3]. Mesenchymal stem cells (MSCs) are multipotent stem cells with the ability to self-renew and have been isolated from various tissues, such as the bone marrow [4], heart [5], adipose [6], peripheral blood [7], dental pulp [8], cord blood [9], menstrual blood [10–12], Wharton' s jelly [13], and chorionic villi [14]. They have the potential to differentiate into the bone, cartilage, fat, and muscle [15, 16]. Therefore, MSCs are a promising source for the cellular treatment of a variety of congenital diseases. However, allogeneic MSC treatment for congenital diseases has generally shown limited long-term engraftment after transplantation [17, 18].

In contrast to the postnatal environment, the fetal environment contains numerous characteristics that may allow for the improvement of stem cell-based therapies; therefore, prenatal cellular transplantation is a promising approach for treating a variety of congenital anomalies. The fetal environment is advantageous for stem cell engraftment because it is naturally receptive to remodelling and regeneration of fetal tissues by stem cells and it is highly conducive to expansion of stem cell compartments [19–23]. Ideally, transplanting autologous fetal stem cells should endow long-term engraftment, even after the baby is born [19, 20, 24]. In consideration of autologous fetal cell sources, however, collection of fetal blood and tissues is technically challenging due to the risk of fetal demise [25–27] and the routine availability of amniocentesis only in the second trimester [28–30]. The placenta is a promising autologous MSC source [31, 32], as chorionic villus sampling (CVS) can be performed in early gestation to obtain fetal stem cells. It has been shown that first trimester fetal MSCs possess several advantages for regenerative medicine over adult and perinatal MSCs [33–37]. Therefore, isolation of MSCs from first trimester chorionic villus tissue (CV-MSCs) that allows for therapeutic use for *in utero* applications represents a promising approach for autologous fetal treatment of birth defects [38].

In our previous studies, we have successfully established the CV-MSC isolation protocol [39] and treated some fetal diseases using CV-MSCs, such as spina bifida [40–42] and hemophilia [43, 44]. However, another limitation to the stem cell therapeutic efficiency is the poor survival of transplanted cells in ischemic target tissue [45, 46]. Most implanted cells may die within several days after transplantation, partially due to the drastic environmental changes [47]. Thus, improving cell engraftment efficiency after transplantation is critical for enhancing stem cell therapeutic efficiency. Several strategies have been designed to solve this problem, such as preconditioning of the cells by oxidative stress, heat shock, and hypoxia [48]. In all of these instances, hypoxic preconditioning (HP) is the best approach to protect stem cells from ischemic damage in animal models [49, 50] and has also been shown to increase protective effects of MSCs on different types of ischemic target tissue [51–54]. However, whether hypoxic preconditioning could enhance autologous CV-MSC-based treatment of fetal diseases has not yet been determined.

Angiogenesis is also essential for tissue development, maintenance, and regeneration to improve therapeutic efficiency in fetal regeneration [55]. Angiogenesis can be stimulated by multiple angiogenic cytokines, such as vascular endothelial growth factor (VEGF), brain-derived neurotrophic factor (BDNF), and hepatocyte growth factor (HGF), secreted from transplanted MSCs [56]. Thus, enhancing the proangiogenic ability of transplanted CV-MSCs will further improve the therapeutic effects of the transplanted cells. In this study, to further improve the therapeutic potential of CV-MSCs for the treatment of fetal diseases, we aimed to develop an optimal HP protocol to enhance survival and proangiogenic capacity of transplanted autologous CV-MSCs for future clinical applications, in treating a variety of fetal diseases.

## 2. Materials and Methods

### 2.1. Cell Isolation and Culture

Human placental tissues (*n* = 4) from the first trimester gestation (≤12 weeks of gestation) were collected from healthy consenting patients during elective abortions at the UC Davis Medical Center, with approval from the Institutional Review Board. CV-MSCs were isolated from chorionic villus tissue using an explant culture method previously established in our lab [39, 41, 57–59]. Chorionic villus tissue was washed in phosphate-buffered saline (PBS, Cat. No.: SH3025601, HyClone) containing 100 U/mL penicillin and 100 *μ*g/mL streptomycin (1% pen-strep, Cat. No.: 15070063, Thermo Fisher Scientific) and dissected into smaller pieces. Tissues were evenly spread across tissue culture-treated flasks and cultured in D5 media containing high-glucose DMEM (Cat. No.: SH3028401, HyClone), 5% fetal bovine serum (FBS, Cat. No.: SH30071.03, HyClone), 20 ng/mL recombinant human basic fibroblast growth factor (bFGF, Cat. No.: 233-FB, R&D systems), and 20 ng/mL recombinant human epidermal growth factor (EGF, Cat. No.: 236-EG, R&D systems) and incubated at 37°C, 5% CO_2_. Cells were allowed to migrate from the tissue and grow to 80-90% confluency before the first passage. The media was changed every 3-4 days. CV-MSCs were used between P3 and P5 for all experiments.

### 2.2. Hypoxic Preconditioning and Simulated Ischemia

**Figure 1:**
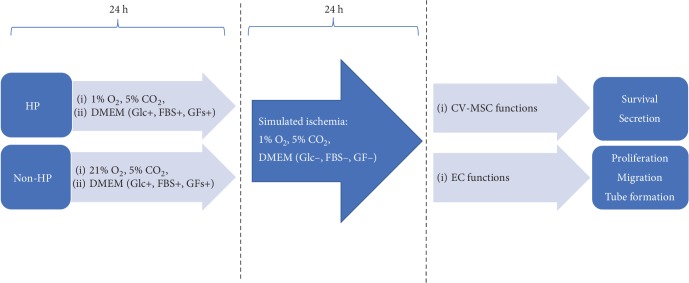
The whole experimental process. CV-MSCs were treated with HP or non-HP for 24 h, then the pretreated CV-MSCs were transferred to a simulated ischemic environment for another 24 h. The survival and secretion of CV-MSC were determined. The effects of condition media obtained from CV-MSCs after ischemic stimulus on EC proliferation, migration, and differentiation were evaluated.

The whole experimental process was depicted in Figure 1. For HP, CV-MSCs were subjected to a hypoxic condition with 1% O_2_ and 5% CO_2_, achieved by replacing O_2_ with N_2_ in an O_2_- and CO_2_-controlled multigas incubator (Coy Laboratory Products Inc.), while being kept in D5 media. CV-MSCs were incubated under normoxic conditions with 21% O_2_ and 5% CO_2_ in D5 media serving as control (non-HP). For simulated ischemia, CV-MSCs were subjected to the 1% O_2_, 5% CO_2_ in glucose-free DMEM (Cat. No.: A14430-01, Thermo Fisher Scientific) without FBS or growth factors which has been widely used for *in vitro* studies to mimic the ischemic environment *in vivo* [60, 61]. After ischemic stimulus, the cells were detached using TrypLE (Cat. No.: 12563029, Gibco), and total cell number was determined using Trypan Blue (Cat. No.: 15250061, Thermo Fisher Scientific). Total DNA was determined using the Quant-iT PicoGreen dsDNA kit (Cat. No.: P7589, Thermo Fisher Scientific). Cell nucleus was stained by DAPI (4′,6-diamidino-2-phenylindole dihydrochloride) (Cat. No.: D3571, Thermo Fisher Scientific) and imaged using an Olympus IX81 microscope. Nuclear area was quantified using ImageJ software (NIH). Cell viability test was performed using a LIVE/DEAD® Fixable Aqua Dead Cell Stain Kit (Cat. No.: L34957, Thermo Fisher Scientific), and the results were imaged using the Olympus IX81 microscope and quantified using ImageJ software.

### 2.3. Flow Cytometry Analysis

CV-MSCs at passage 5 were detached using Accutase (Cat. No.: A1110501, Thermo Fisher Scientific) and divided into 1 × 10^6^ cells per sample for assessment by flow cytometry. Expression of surface markers was analyzed using the following antibodies: FITC-CD44 (Cat. No.: 560977, clone G44-26), PE-CD90 (Cat. No.: 561970, clone 5E10), PE-CD73 (Cat. No.: 561014, clone AD2), APC-CD105 (Cat. No.: 562408, clone 266), APC-CD29 (Cat. No.: 561794, clone MAR4), PE-CD34 (Cat. No.: 550761, clone 563), PE-CD31 (Cat. No.: 560983, WM59), APC-CD45 (Cat. No.: 560973, clone HI30), and appropriate isotype controls including PE-Ms IgG1 k (Cat. No.: 556650, clone MOPC-21), APC-Ms IgG1 k (Cat. No.: 550854, clone MOPC-21), and FITC-Ms IgG2b k (Cat. No.: 556655, clone 27-35) (all from BD Biosciences). Cells were fixed in 10% formalin (Cat. No.: SF-100, Thermo Fisher Scientific) for 30 minutes prior to analysis and were analyzed on a BD LSRFortessa cell analyzer, and further data analysis and gating were performed using FlowJo software (FlowJo, LLC). The flow cytometry analysis gating strategy was as follows: CV-MSCs were based on size and density from forward and side scatter. From the CV-MSC population, single cells were then gated based on forward scatter height verse forward scatter area. From the single cell populations of CV-MSCs, live cells were further gated using the exclusion viability dye NearIR (Cat. No.: L34975, Thermo Fisher Scientific). Phenotypes were determined using isotype controls for each respective fluorophore. Gating on isotypes was determined to be less that 1 percent positive.

### 2.4. Evaluation of CV-MSC Survival

CV-MSCs were seeded in 96-well plates (15,000 cells/cm^2^) with three independent samples in duplicates. After HP and ischemic stimulus, the cells were lysed and analyzed by caspase-3 assay using a Caspase-3 Activity Assay Kit (Cat. No.: 5723, Cell Signaling Technology) according to the manufacturer's instruction. Fluorescence (ex 380 nm/em 450 nm) was measured using a SpectraMax i3x Multi-Mode Detection Platform (Careforde Safety & Scientific). The cells also were determined using a CellTiter 96® AQueous One Solution Cell Proliferation Assay (MTS, Cat. No.: G3582, Promega) according to the manufacturer's instruction. The amount of soluble formazan product produced by the reduction of MTS by metabolically active cells was measured at the 490 nm absorbance using the SpectraMax i3x Multi-Mode Detection Platform.

### 2.5. Western Blot Analysis

After HP and ischemic stimulus, cells were washed with DPBS and lysed in lysis buffer containing RIPA lysis buffer (Cat. No.: 89901, Thermo Fisher Scientific), sodium metavanadate (Cat. No.: sc-251034, NaVO_3_, Santa Cruz Biotechnology), PMSF (Cat. No.: 36978, Thermo Fisher Scientific), and protease and phosphatase inhibitor cocktail (Cat. No.: 78440, Thermo Fisher Scientific). Protein concentration was determined by bicinchoninic acid (BCA) protein assay (Cat. No.: 23227, Thermo Scientific); denatured protein was resolved in a NuPAGE™ 4-12% Bis-Tris Protein Gel (Cat. No.: NP0323, Thermo Fisher Scientific) and transferred to a nitrocellulose membrane (Cat. No.: 77012, Thermo Fisher Scientific). Membranes were blocked and incubated overnight at 4°C with primary antibodies against AKT (Cat. No.: 9272, Cell Signaling Technology), phospho-AKT (Cat. No.: 4060, Cell Signaling Technology), and GAPDH (Cat. No.: sc-32233, Santa Cruz Biotechnology). Subsequently, membranes were incubated for 1 h with conjugated secondary antibodies (Cat. No.: 7074, Cell Signaling Technology) at room temperature and blots were imaged using a ChemiDoc MP^+^ imaging system (Bio-Rad), and further data analysis and gating were performed using ImageJ software.

### 2.6. Cytokine Secretion of CV-MSCs

To determine the effect of the hypoxic condition on CV-MSC secretion, CV-MSCs were seeded in 96-well plates (15,000 cells/cm^2^) with three independent samples in duplicates and cultured in D5 media under hypoxic condition with 1% O_2_ or normoxic condition with 21% O_2_. To determine the effect of HP on CV-MSC secretion under ischemic stimulus, CV-MSCs were seeded in 96-well plates (15,000 cells/cm^2^) with three independent samples in duplicates and cultured in D5 media under hypoxic condition with 1% O_2_ or normoxic condition with 21% O_2_ for 24 h. The cells were then transferred and subjected to the 1% O_2_ in glucose-free DMEM without FBS or growth factors. Supernatants were collected and analyzed by ELISA using individual VEGF (Cat. No. DVE00, R&D Systems), HGF (Cat. No. DHG00B, R&D Systems), or BDNF (Cat. No. DBD00, R&D Systems) ELISA kit according to the manufacturer's instructions. The data was collected at 450 nm absorbance using a SpectraMax i3x Multi-Mode Detection Platform.

### 2.7. Proangiogenic Capacity of CV-MSCs

Conditioned media collected from CV-MSCs with HP or non-HP after ischemic stimulus were centrifuged at 10000 rpm for 10 min to remove floating cells and debris. For survival experiments, human endothelial colony forming cells (HECFCs) were seeded in 96-well plates (20000 cells/cm^2^) with three independent samples in duplicates and cultured in the conditioned media for 5 days. The media were changed every other day. The viable cell number was determined at different time points using MTS assay. For migration experiments, HECFCs were seeded in Culture-Insert 2 Well in *μ*-Dish (Cat. No.: 81176, ibidi) with three independent samples in duplicates and cultured in the conditioned media for 12 h. Images were taken using a Carl Zeiss Axio Observer D1 inverted microscope. The cell-covered area was quantified using ImageJ software. For tube formation experiments, HECFCs were seeded in 96-well plates precoated with 50 *μ*L Matrigel (Cat. No.: 354234, Corning) according to the manufacturer's instructions at a density of 10^4^ cells/well with three independent samples in duplicates and cultured in the conditioned media. After 12 h, cells were observed using the Carl Zeiss Axio Observer D1 inverted microscope. The total segment tube length was quantified using ImageJ software.

### 2.8. Statistical Analysis

For two-sample comparison, Student's *t*-test was used. For multiple-sample comparison, the significance of intergroup differences was tested by one-way analysis of variance (ANOVA), and Tukey's multiple comparisons test was used for postanalysis. A *p* value of 0.05 or less indicates significant difference between samples in comparison.

## 3. Results

### 3.1. HP Protected CV-MSCs from Ischemic Damage

**Figure 2:**
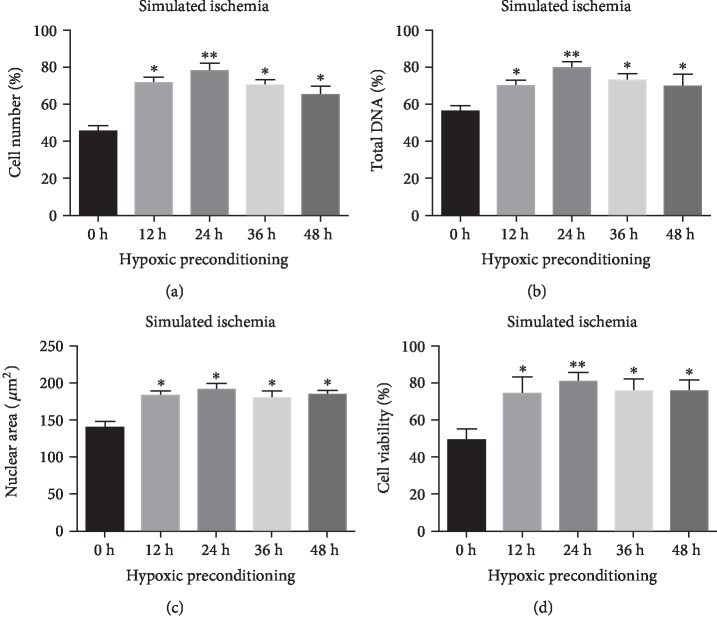
HP reduced ischemic damage of CV-MSCs. Cell number (a), total DNA (b), nuclear area (c), and cell viability (d) of CV-MSCs pretreated with HP or non-HP for 0 h, 12 h, 24 h, 36 h, or 48 h and followed by 24 h cultivation under ischemic stimulus. Data are expressed as mean ± standard deviation: ^∗^*p* < 0.05 and ^∗∗^*p* < 0.01 (*n* = 4).

Prior to ischemic stimulus, CV-MSCs with HP and CV-MSCs with non-HP were, respectively, cultured in 1% O_2_ and D5 medium or 21% O_2_ and D5 medium for 12 h, 24 h, 36 h, or 48 h. Both CV-MSCs with HP and CV-MSCs with non-HP were then cultured in the ischemic environment for 24 h. In addition to cell viability, alteration in nuclear morphology is another important indication in apoptotic cells. The nuclear morphology in injured or dead cells generally falls into nuclear condensation: a decrease in nuclei size without chromatin margination [62]. In this study, we evaluated the cell viability using three different approaches and assessed the nuclear morphology by DAPI staining and fluorescence microscopy. The results of remaining viable cell number (Figure 2(a)), total DNA (Figure 2(b)), nuclear area (Figure 2(c)), and cell viability (Figure 2(d)) uniformly showed that ischemic damage of CV-MSCs was significantly reduced after shorter or longer HP periods, and the 24 h HP proved the most efficient. Statistical analysis was performed between each group, and the results showed significant differences among all the hypoxic preconditioning groups (12 h, 24 h, 36 h, and 48 h) and the control group (0 h), but no significant differences among the hypoxic preconditioning groups (12 h, 24 h, 36 h and 48 h).

### 3.2. HP Did Not Alter the Immunophenotype of CV-MSCs

**Figure 3:**
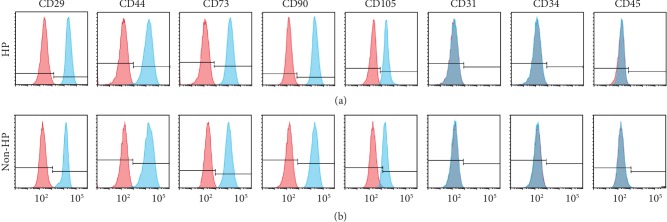
Immunophenotype of CV-MSCs with HP and CV-MSCs with non-HP. Flow cytometry results displayed profiles of both the CV-MSCs with HP (a) and the CV-MSCs with non-HP (b) which were positive for markers CD29, CD44, CD73, CD90, and CD105, whereas they were negative for markers CD31, CD34, and CD45.

In order to evaluate if HP altered the immunophenotype of CV-MSCs, we characterized the CV-MSCs cultured in the hypoxic environment (1% O_2_, DMEM with glucose, FBS, and GFs) for 24 h (CV-MSCs with HP) and the CV-MSCs cultured in the normoxic environment (21% O_2_, DMEM without glucose, FBS, and GFs) for 24 h (CV-MSCs with non-HP) by using flow cytometry analysis, which was shown in the first 24 h section in the diagram (Figure 1). The results displayed profiles of both the CV-MSCs with HP and the CV-MSCs with non-HP which were positive for well-established MSC markers CD29, CD44, CD73, CD90, and CD105 [63], whereas they were negative for hematopoietic and endothelial-related markers CD31, CD34, and CD45 (Figure 3). The percentage of immunophenotype of CV-MSCs with HP and CV-MSCs with non-HP is shown in Table 1. There is no significant difference between the two groups. These results demonstrated that HP did not alter the immunophenotype of CV-MSCs.

### 3.3. HP Reduced Apoptosis of CV-MSCs under Ischemic Stimulus via Activated Related Biological Signals

**Figure 4:**
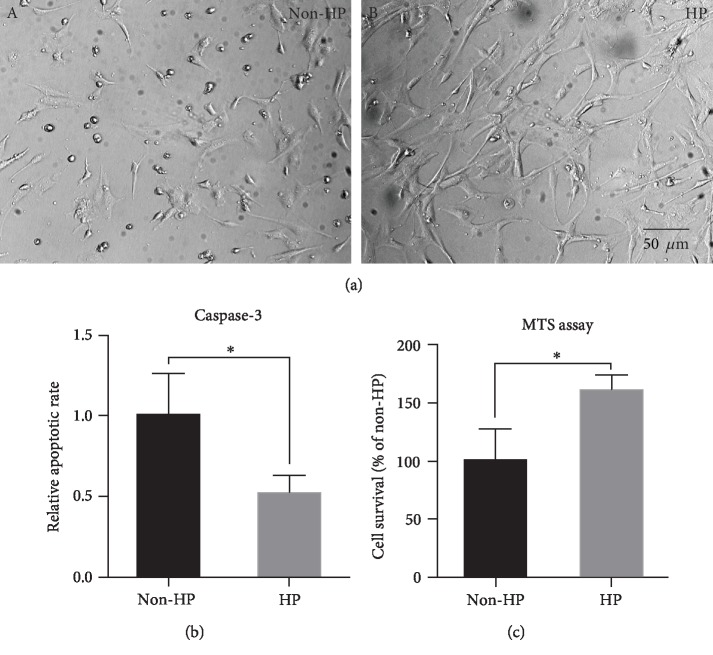
HP reduced the apoptosis of CV-MSCs after ischemic stimulus. Representative microphotographs taken by light microscopy (a), caspase-3 activity (b), and MTS assay (c) of CV-MSCs with HP or CV-MSCs with non-HP after ischemic stimulus for 24 h. Data are expressed as mean ± standard deviation: ^∗^*p* < 0.05 (*n* = 4).

**Figure 5:**
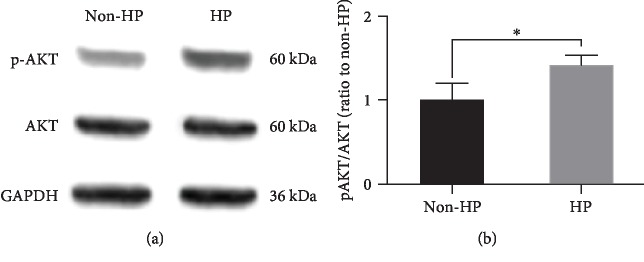
Effects of HP on CV-MSC biological functions after ischemic stimulus. Western blot analysis of AKT (60 kDa), p-AKT (60 kDa), and GAPDH (36 kDa) expressed in CV-MSCs with HP or CV-MSCs with non-HP (a) after ischemic stimulus for 24 h. Quantification and correlative statistical analysis (b). Data are expressed as mean ± standard deviation: ^∗^*p* < 0.05 (*n* = 4).

To determine the effect of HP on CV-MSC apoptosis under ischemic stimulus, microphotographs taken by light microscopy showed that CV-MSCs with HP (Figure 4(a), B) were healthier than CV-MSCs with non-HP after ischemic stimulus (Figure 4(a), A). Caspase-3 assay and MTS assay were performed to further confirm the effect of HP on CV-MSC apoptosis after ischemic stimulus. The results showed that caspase-3 activity in CV-MSCs with HP was significantly less than that measured in CV-MSCs with non-HP (Figure 4(b)). The MTS assay results showed HP significantly enhanced CV-MSC survival after ischemic stimulus (Figure 4(c)). The activation of AKT does not only inhibit the proapoptotic factors but also activates the transcription of antiapoptotic genes, which are involved in the regulation of the key cellular functions including cell growth and survival [64]. Our subsequent Western blot data showed that the activated form of AKT, phosphorylated-AKT (p-AKT), expressed in CV-MSCs with HP was significantly higher compared to that expressed in CV-MSCs with non-HP after ischemic stimulus (Figures 5(a) and 5(b)).

### 3.4. HP Enhanced Angiogenic Growth Factor Release of CV-MSCs under Ischemic Stimulus

**Figure 6:**
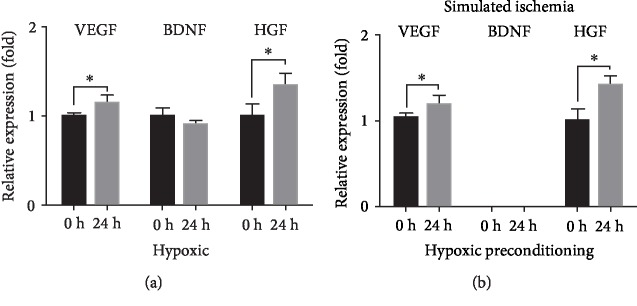
Growth factor release of CV-MSCs in hypoxic condition and effect of HP on growth factor release of CV-MSCs under ischemic stimulus. Effect of hypoxic condition on growth factor release of CV-MSCs (a). Effect of HP on growth factor release of CV-MSCs cultured under ischemic stimulus for 24 h (b). Data are expressed as mean ± standard deviation: ^∗^*p* < 0.05 (*n* = 4).

VEGF, BDNF, and HGF play important roles in angiogenesis [65–68]. In this study, the ELISA test was performed to determine the angiogenic growth factor release of CV-MSCs. For the effect of the hypoxic condition on the growth factor release of CV-MSCs, the results showed that the release of VEGF and HGF from CV-MSCs cultured in D5 media were significantly higher in the hypoxic condition compared to the normoxic condition and that there was no significant difference for BDNF release (Figure 6(a)). For the effect of HP on the growth factor release of CV-MSCs cultured under ischemic stimulus for 24 h, the results showed that the release of VEGF and HGF by CV-MSCs in the HP group was significantly higher compared to that in the non-HP group and that BDNF could not be determined in either HP or non-HP groups (Figure 6(b)).

### 3.5. HP Enhanced the Proangiogenic Properties of CV-MSCs under Ischemic Stimulus

**Figure 7:**
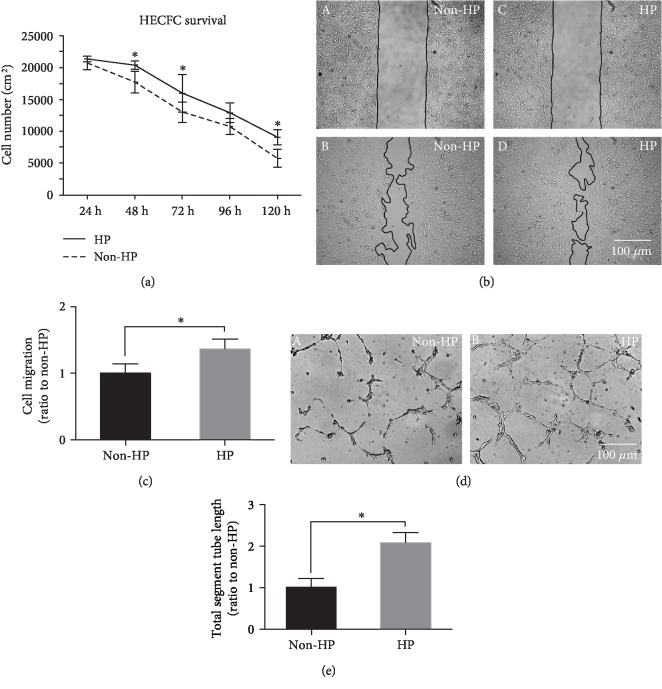
Effects of HP on CV-MSC proangiogenic properties under ischemic stimulus. After ischemic stimulus for 24 h, conditioned media collected from CV-MSCs with HP significantly enhanced HECFC survival (a), migration (b, c), and tube formation (d, e) compared to conditioned media collected from CV-MSCs without HP. The data were quantified, and statistical analyses were performed. Data are expressed as mean ± standard deviation: ^∗^*p* < 0.05 (*n* = 4).

The conditioned media collected from CV-MSCs with HP or CV-MSCs without HP cultured in ischemic stimulus for 24 h were used for angiogenic property evaluation. The MTS assay results showed that survival of HECFCs cultured in the conditioned media collected from CV-MSCs with HP was significantly enhanced compared to the survival of HECFCs cultured in the condition media collected from CV-MSCs without HP, and viable cell numbers in both groups decreased over time (Figure 7(a)). The migration and tube formation results showed that the conditioned media collected from CV-MSCs with HP significantly improved HECFC migration (Figures 7(b) and 7(c)) and tube formation (Figures 7(d) and 7(e)) compared to the condition media collected from CV-MSCs without HP.

## 4. Discussion

Fetal diseases, which can be devastating and burdensome for afflicted children and their families, have attracted much attention in recent decades [69]. Fetal surgery has progressed and been performed as an effective method of therapy for select fetal diseases [70, 71]. Stem cell-based therapy has emerged as a promising area of research to enhance treatment for a variety of diseases [72, 73] and has been used extensively to augment existing *in utero* surgical techniques for fetal diseases [74, 75]. To further promote the clinical application of stem cell-based therapy in fetal medicine, practical approaches must be established to overcome the general issues associated with cell therapy, such as limited *ex vivo* expansion capacity, low multipotent differentiation ability, inflammatory reactions, immunosuppression, and low cell survival and engraftment. Autologous fetal stem cells isolated from fetal tissues, such as placenta and amniotic fluid, possess an intermediate phenotype between embryonic and adult cells, multipotent differentiation ability, anti-inflammatory properties, and low immunogenicity that make them an ideal candidate for fetal regenerative medicine [76, 77]. Hence, cell survival and engraftment after transplantation is a major challenge to the success of *in utero* stem cell transplantation.

HP refers to the exposure of cells to a moderate hypoxic environment that results in a resistance to subsequent severe ischemic damage for cells. HP has been shown to protect and improve MSC functions after transplantation [78–80]. In this study, we used 1% oxygen for CV-MSC preconditioning and simulated an ischemic environment to mimic the low oxygen concentration in the fetal environment and the further decrease in local oxygen concentration present after cell transplantation [81, 82]. The results of cell viability and alterations in nuclear morphology showed HP protected CV-MSCs from ischemic damage. HP has also been demonstrated to increase the expression of endogenous regenerative factors in stem cell therapy [83–85]. The AKT pathway is essential for cell survival, as activated AKT influences many factors involved in apoptosis, either by transcription regulation or by direct phosphorylation [86]. In this study, we demonstrated that the activated form of AKT, phosphorylated-AKT, expressed in CV-MSCs with HP is significantly higher compared to that expressed in CV-MSCs without HP. These results further support our previous results that HP significantly reduces CV-MSC apoptosis under ischemic stimulus. Previous studies have demonstrated that MSC transplantation provides many benefits for the treatment of various diseases [87–89]. One advantage is that they secrete a variety of cytokines that exert general protective and regenerative effects *in vivo*, such as immunomodulation and tissue regeneration [90, 91]. HP has been confirmed to enhance the paracrine effects of MSCs and has also been applied for several diseases [92]. Our previous work showed that CV-MSCs robustly secrete growth factors, cytokines, and angiogenic proteins, such as VEGF, BDNF, and HGF [39]. In this study, we evaluated the effect of HP on CV-MSC secretion and demonstrated that secretion of several cytokines related to vascular protection, such as VEGF and HGF, was significantly higher in the HP group, compared to the non-HP group under ischemic stimulus. These results indicate that HP of CV-MSCs could effectively promote tissue repair and formation of new blood vessels in the surrounding tissue, which is consistent with previous studies of different types of MSCs [92, 93]. According to the results, we also found that the environmental nutrition level was critical for the release of BDNF which is another angiogenic growth factor that can promote endothelial cell survival and induce angiogenesis in ischemic tissues [68].

Regenerated tissues of a clinically relevant size need a robust vascular network to supply nutrients and oxygen. To supply all cells with sufficient nutrients, and to successfully connect the regenerated vasculature to the existing vasculature, the newly formed vascular network needs to be highly organized [94, 95]. VEGF is a key contributor in the formation of new blood vessels, which can induce growth of preexisting (angiogenesis) or *de novo* vessels (vasculogenesis) and is therefore key for embryonic development and vessel repair [65, 66]. HGF is a potent angiogenic factor that stimulates endothelial cell motility and growth, which also enhances VEGF-induced angiogenesis [67]. Based on these findings, we evaluated the effect of HP on EC functions and showed that HP significantly enhances the proangiogenic properties of CV-MSCs under ischemic stimulus, by improving the secretion of VEGF and HGF. Hence, transplanting CV-MSCs primed with HP represents an ideal approach for fetal tissue engineering and treatment of various fetal diseases.

## 5. Conclusions

In this study, we demonstrated that HP significantly enhances the survival, secretion of bioactive factors, and proangiogenic capacity of CV-MSCs under ischemic stimulus. Based on our previous work demonstrating that early gestation CV-MSCs are an ideal cell source for fetal tissue engineering and disease treatment, this study successfully developed an optimal and practical approach to expand and improve the efficiency of applying CV-MSCs for fetal tissue engineering and treatment of various fetal diseases.

## Figures and Tables

**Table 1:** Percentage of immunophenotype of CV-MSCs with HP and CV-MSCs with non-HP.

	CD29	CD44	CD73	CD90	CD105	CD31	CD34	CD45
HP	99.6 ± 0.08%	99.0 ± 0.1%	99.4 ± 0.03%	99.8 ± 0.06%	98.5 ± 0.08%	0.12 ± 0.02%	0.12 ± 0.01%	0.16 ± 0.03%
Non-HP	99.4 ± 0.1%	98.6 ± 0.1%	99.2 ± 0.1%	99.6 ± 0.1%	98.0 ± 0.1%	0.12 ± 0.03%	0.11 ± 0.01%	0.12 ± 0.02%
*p* value	0.7	0.6	0.7	0.7	0.4	0.7	0.6	0.2

## Data Availability

The data used to support the findings of this study are available from the corresponding author upon request.
